# Flexible Receiver Antenna Prepared Based on Conformal Printing and Its Wearable System

**DOI:** 10.3390/s25144488

**Published:** 2025-07-18

**Authors:** Qian Zhu, Wenjie Zhang, Wencheng Zhu, Chao Wu, Jianping Shi

**Affiliations:** School of Electrical and Automation Engineering, Nanjing Normal University, Nanjing 210046, China; 231812114@njnu.edu.cn (Q.Z.); 231812074@njnu.edu.cn (W.Z.); 241802017@njnu.edu.cn (W.Z.)

**Keywords:** microwave energy, conformal printing, flexible antenna, wearable device

## Abstract

Microwave energy is ideal for wearable devices due to its stable wireless power transfer capabilities. However, rigid receiving antennas in conventional RF energy harvesters compromise wearability. This study presents a wearable system using a flexible dual-band antenna (915 MHz/2.45 GHz) fabricated via conformal 3D printing on arm-mimicking curvatures, minimizing bending-induced performance loss. A hybrid microstrip–lumped element rectifier circuit enhances energy conversion efficiency. Tested with commercial 915 MHz transmitters and Wi-Fi routers, the system consistently delivers 3.27–3.31 V within an operational range, enabling continuous power supply for real-time physiological monitoring (e.g., pulse detection) and data transmission. This work demonstrates a practical solution for sustainable energy harvesting in flexible wearables.

## 1. Introduction

Flexible wearable devices have advanced significantly in recent decades [1,2], with applications spanning diverse aspects of daily life [3,4]. However, the growing sophistication of wearable electronics presents new challenges in energy supply. Most current devices rely on wired charging, which introduces usability constraints [5], while many wireless charging systems require close proximity to the power source, severely limiting user mobility during charging [6]. To address these limitations, harvesting ambient energy has emerged as a promising solution for powering wearable devices. Researchers have explored various energy sources, including mechanical pressure [7], light [8], and electromagnetic (EM) waves [9]. With the rapid development of wireless communication technologies, our environment is now saturated with radio-frequency (RF) signals from base stations, Wi-Fi routers, and other devices. Rectennas (rectifying antennas) can convert these ambient EM waves into usable direct current (DC) power, making them a widely studied solution for energy harvesting [10,11,12,13]. In wearable devices, radio-frequency (RF) energy harvesters predominantly operate in the low-to-medium frequency bands, with each band offering distinct advantages [14]: 433 MHz (ISM band) exhibits strong penetration capability, making it suitable for short-range, high-efficiency energy harvesting; 868 MHz (Europe)/915 MHz (North America, ISM bands) balances transmission range and power conversion efficiency, ideal for wide-area energy scavenging; and 2.45 GHz (global ISM band) benefits from abundant ambient RF energy, rendering it advantageous for high-density wireless power transfer applications.

Operating frequency selection involves inherent trade-offs in wearable antenna design: lower frequencies increase antenna dimensions due to wavelength constraints, potentially compromising device compactness, while higher frequencies exhibit greater path loss, reducing power transfer efficiency. Through a comprehensive analysis of these competing factors, this work strategically selects 915 MHz and 2.45 GHz as the operational frequencies to achieve balance between the wearable form factor and RF energy harvesting performance. While ambient EM energy is abundant, its power density is often unstable and unpredictable. To ensure reliable system operation, this study proposes a hybrid approach: combining ambient RF energy harvesting with a dedicated RF transmitter. This dual-source strategy enhances energy availability and ensures stable power delivery for low-power wearable applications.

Prior research has developed numerous RF energy harvesting systems [15,16,17,18], which can be classified into two categories: rigid substrate-based and flexible substrate-based systems. For rigid systems, Wei et al. [19] designed a hybrid energy harvester combining a high-efficiency transparent antenna, rectifier circuits, and solar cells. While this system achieves high output by integrating a micro-grid antenna with solar panels, its rigid substrate and large size make it unsuitable for wearable applications. Flexible substrate systems have received less attention. Bu et al. [20] proposed a wearable-compatible design using a transparent antenna on a PDMS substrate. However, this approach has limitations: the antenna was simulated and fabricated on a flat surface, meaning its performance degrades when bent during actual wearable use, reducing energy transmission efficiency.

To address these challenges, this paper presents a novel flexible receiving antenna fabrication method based on conformal 3D printing (CDP) technology, along with a corresponding wearable device system. The proposed approach leverages CDP’s multi-axis synchronized nozzle system, enabling precise 3D structure fabrication on complex curved surfaces [21]. This technique not only overcomes the geometric limitations of conventional manufacturing but also ensures a safe, non-damaging printing process for the wearer. By integrating 3D body scanning, the system achieves personalized wearable device customization, where the optimized fit minimizes performance deviations caused by antenna deformation and enhances wireless power transfer efficiency. The developed device incorporates a flexible antenna-based wireless power module, enabling long-term, continuous physiological signal monitoring. Combining advanced manufacturing with biomedical engineering, this solution ensures wearability and system stability, opening up a new avenue for the application of RF energy harvesting technology in medical wearable devices.

## 2. System Overview

**Figure 1:**
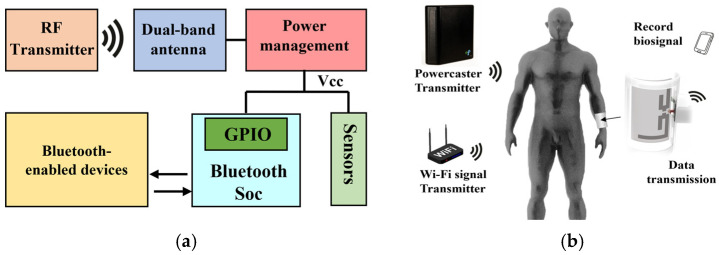
(**a**) System working schematic diagram; (**b**) system working status.

The working principle of the whole system is shown in Figure 1a. The system adopts a dual-band cooperative power supply architecture, which integrates a commercial 915 MHz RF transmitter with ambient 2.45 GHz Wi-Fi routers to establish a hybrid energy field. This configuration, combined with an adaptive impedance-matching network and high-efficiency rectifying circuits, enables stable power delivery to wearable terminals.

The core innovation of this design lies in the use of advanced conformal 3D printing technology to prepare the flexible receiving antenna, and through an antenna structure design that accurately matches the curved surface of the human body, the parameter shift caused by the deformation of the traditional flexible antenna is effectively suppressed, and the energy transmission efficiency on the skin surface is significantly improved. Different frequency bands of energy harvesting devices and capacitive energy storage are designed to optimize energy management, in order to maintain a stable operating voltage over a wide range of distances and achieve stable operation of the device. Real-time data communication and sensor digitization are facilitated by a Bluetooth (BLE) low-power system on a chip (SoC). In addition, the system adopts a modular design, allowing users to flexibly configure the functional modules according to their needs and achieve personalized customization.

The working state of the device is shown in Figure 1b. The system uses wireless energy transmission technology to power the functional modules on the surface of the human body through an RF transmitter. The sensing module collects the corresponding data in real time and then transmits the data to the preset mobile terminal via the Bluetooth module. The mobile terminal application visualizes the data so that the user can intuitively grasp the real-time physiological parameter changes.

## 3. Design and Fabrication of Flexible Antenna

In order to improve the transmission efficiency of RF energy, this study adopts conformal printing technology to prepare a curved flexible antenna. Prior work by our research group has demonstrated substantial progress in conformal printing technologies [22]. The conformal printing process of the flexible receiving antenna includes the following steps: Firstly, a 3D scanner is used to obtain the 3D data of the wearing part; subsequently, the curved substrate matching the structure of the human arm is prepared based on the data, and then the antenna structure is accurately mapped on the curved surface through path reprogramming. Finally, a traditional three-axis 3D printer is modified to directly print the flexible receiving antenna according to the generated surface trajectory.

**Figure 2:**
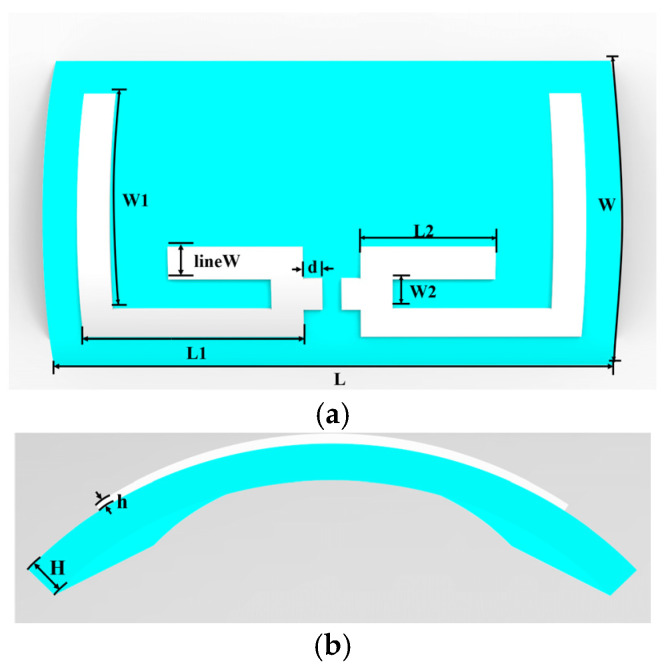
(**a**) Front view of antenna structure; (**b**) side view of antenna structure.

In this study, the curvature data of the wearer’s wrist is obtained by 3D scanning, and its geometric features are equivalent to an elliptic cylindrical surface model with a long axis of 60 mm and a short axis of 33.8 mm, which is used as a conformal substrate for the flexible antenna. In order to meet the omnidirectional reception requirements during human activities [23], an omnidirectional dipole antenna design with a symmetric dual-vibrator structure is proposed. The design adopts the structural optimization method of bending the high-frequency vibrator, which effectively reduces the lateral size of the antenna. In the simulation and analysis stage, the elliptic cylindrical surface model is firstly imported into the Computer Simulation Technology Studio Suite (CST) as a conformal substrate, and the antenna structure is optimized by parametric scanning. In order to evaluate the effect of the surface conformal substrate on antenna performance, a planar substrate antenna with the same structure is designed simultaneously as a control. After optimization, the S11 parameters of both antennas work stably in the target frequency bands (915 MHz and 2.45 GHz). Figure 2 shows the final optimized curved flexible antenna structure and its key performance parameters.

The antenna’s critical dimensions are derived from fundamental electromagnetic theory, where the structural parameters are optimized to achieve target resonant frequencies at 915 MHz and 2.45 GHz. The specific relationship is shown in Equation (1). Here, c is the speed of light, $f_{r}$ represents the target resonant frequency, and $\varepsilon_{eff}$ denotes the equivalent relative permittivity of the substrate. The approximate dimensions ($L_{eff}$) of the dipole antenna are calculated accordingly, and then optimized through CST simulations, with the resulting structural parameters listed in Table 1.

(1) $$L_{eff}=\frac{c}{2f_{r\sqrt{\varepsilon_{eff}}}}$$

**Figure 3:**
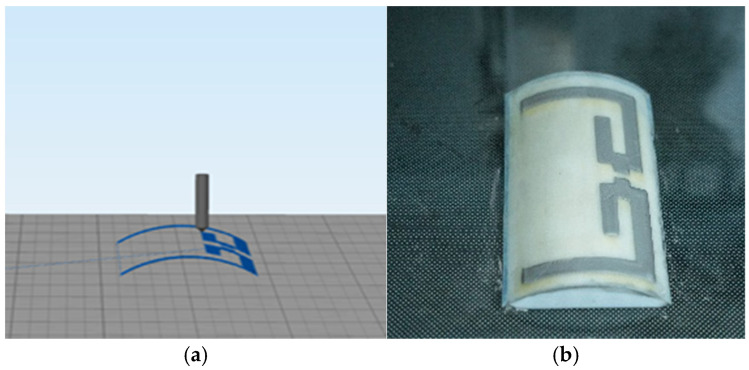
(**a**) The generated conformal trajectories; (**b**) the curved conformal antenna prepared by conformal printing.

In order to achieve the surface conformal preparation of the flexible antenna, this study firstly imports the geometric models of the metal radiating layer and the flexible substrate into the MATLAB (R2023b) software platform. Based on the aforementioned conformal mapping algorithm, the planar antenna structure is accurately converted into a 3D printing path that matches the equivalent wrist elliptic cylindrical surface. Subsequently, the generated conformal trajectory data is imported into the three-axis 3D printing system, and the spatial alignment of the print path is completed by precisely locating the spatial coordinates of the trajectory start point and the surface reference point. As shown in Figure 3a, the print trajectory optimized by the conformal algorithm can accurately fit the target surface. The G-code generated automatically by the algorithm achieves high-precision surface conformal printing on a traditional three-axis printer by precisely controlling the nozzle movement and the displacement of the printing platform. The finally prepared curved dual-frequency dipole antenna is shown in Figure 3b, and the liquid metal conductive layer exhibits good structural integrity and surface fitting, which verifies the feasibility of the proposed conformal printing method. The print preparation process is shown in Video S1.

**Figure 4:**
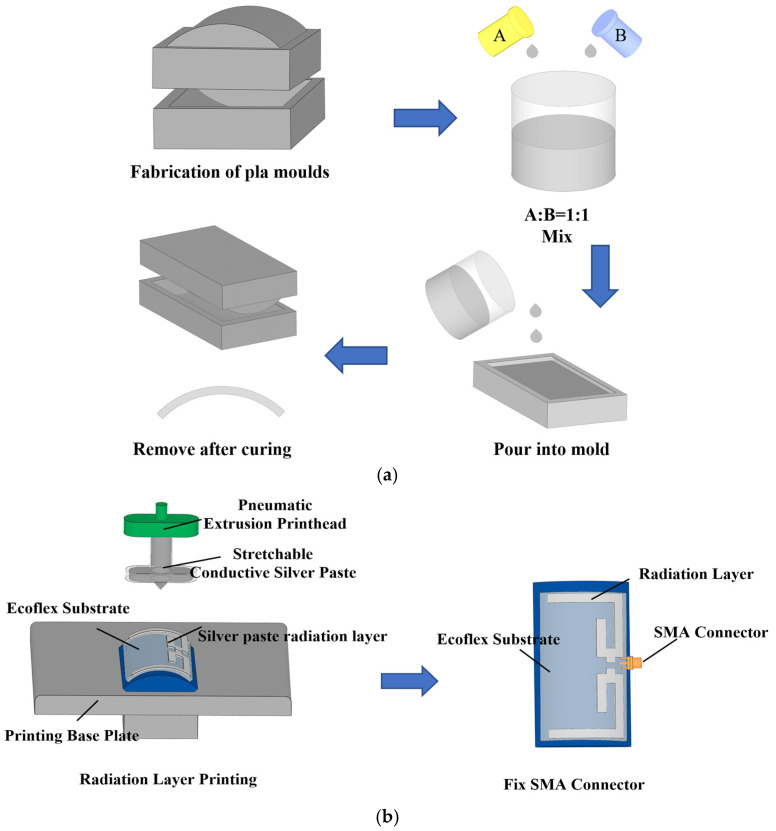
(**a**) Flexible ECOFLEX substrate preparation process; (**b**) flexible antenna production process.

The flexible receiving antenna designed in this study adopts a two-layer structure, including a liquid metal radiating layer and a flexible substrate. Among them, the preparation process of the flexible substrate is shown in Figure 4a, and the specific steps are as follows: ECOFLEX (a class of eco-friendly flexible elastomers) silicone rubber, which has excellent flexibility and biocompatibility, is selected as the substrate material, and it is prepared by an inverted molding process. Firstly, a high-precision Polylactic acid (PLA) mold was processed by fused deposition molding (FDM); subsequently, the two-component ECOFLEX prepolymer was mixed and degassed at a mass ratio of 1:1 and cast into the mold; and finally, the mold was released after room temperature curing to obtain a flexible ECOFLEX substrate with high resilience and mechanical stability. This process effectively ensures the conformal fit of the substrate to the human body surface.

As shown in Figure 4b, the preparation process of the flexible dual-band antenna is mainly divided into three key steps: firstly, a highly conductive silver paste is precisely sprayed on the surface of the ECOFLEX flexible substrate using the conformal printing technique to form the radiating layer structure; subsequently, the silver paste is cured and molded by the thermal curing process to ensure good electrical conductivity and mechanical stability; and lastly, the SubMiniature version A (SMA) connector is integrated to complete the antenna assembly. As a comparison, the preparation of the planar-structure flexible antenna adopts the same process route, only omitting the conformal mapping step. This method ensures the electrical properties and realizes the precise conformal shape of the antenna structure and the curved substrate at the same time.

**Figure 5:**
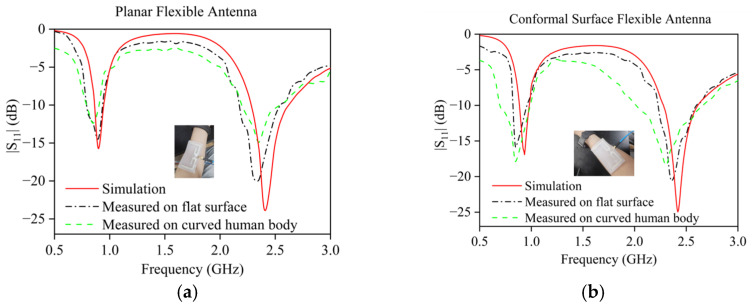
(**a**) Simulation, measurement, and wear comparison of s11 parameters of planar flexible antenna; (**b**) simulation, measurement, and wear comparison plots of s11 parameters of curved conformal flexible antenna.

The omnidirectional half-wave dipole flexible antenna designed in this study exhibits a good consistency of gain in all directions in its radiation characteristics, so we focus on analyzing its S11-parameter characteristics. A vector network analyzer is used to test the S-parameter performance of the flexible antennas with planar and curved structures in the planar state and wearing bending state, respectively, and the test results are shown in Figure 5. Figure 5a demonstrates a comparison of simulated and measured S-parameters for the planar flexible antenna, while Figure 5b presents the corresponding test results for the curved flexible antenna. The test data show that both antennas exhibit good agreement between the measured and simulated results in the planar state. However, the two antennas exhibit significantly different performance characteristics when worn in the bending state. The conformally printed curved antenna exhibits excellent stability: the LF operating point is shifted from 897 MHz to 872 MHz (25 MHz offset) and the HF operating point is shifted from 2.35 GHz to 2.31 GHz (40 MHz offset), and it still maintains a good match (S11 < −10 dB) in the target bands (915 MHz and 2.45 GHz). The planar-prepared flexible antenna shows large performance degradation: the LF operating point is shifted from 904 MHz to 856 MHz (48 MHz offset) and the HF operating point is shifted from 2.37 GHz to 2.29 GHz (80 MHz offset), which results in the significant degradation of matching performance at the 2.45 GHz frequency point (S11 ≈ −9dB). The co-printed curved antenna has a significant advantage in the wearing condition: its frequency offset is reduced by about 50% compared with the planar antenna, and it always maintains good impedance-matching characteristics in the target frequency band. This feature effectively reduces the power loss caused by frequency offset and impedance mismatch, which verifies the practical value of conformal design in wearable applications.

## 4. Design of a Dual-Frequency RF Energy Receiving System

For the RF energy transmission system, the power received by the antenna will change due to the distance from the microwave transmitter, the environmental electromagnetic wave energy, and other factors, and it is difficult to operate in a constant-power situation. Therefore, after the RF signal is received by the antenna, the energy needs to be converted to DC through a suitable rectifier circuit. In the matching circuit, it is difficult for the traditional collector element to ensure the inductance–capacitance value at high frequencies, and the use of a microstrip line structure for matching will also greatly increase the size of the device, which is not conducive to the wearability of the device [24].

**Figure 6:**
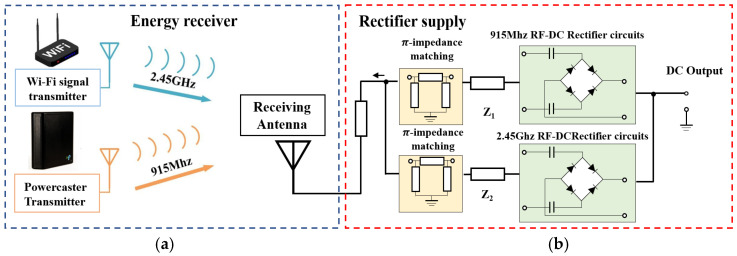
(**a**) Dual-frequency energy reception; (**b**) RF-DC energy conversion.

In order to solve the above problems of general RF energy receiving systems, this paper uses a matching network combining lumped elements and microstrip lines. While ensuring that the circuit size is appropriate, it broadens the range of receiving frequencies and reduces the energy loss caused by environmental factors. The matching circuits of two frequency bands are designed separately, and an independent rectifier channel is designed for each band to reduce the electromagnetic coupling of the two bands, with the microstrip line structure further used to separate the output impedance of the two bands to improve the whole energy receiving system. The designed energy receiving system is shown in Figure 6. The antenna receives signals from two frequency bands at the same time, and the two output impedances are further separated after passing through microstrip line A. The output impedances of the two frequency bands correspond to the input impedances of the two matching networks, and after the two circuits have been π-type impedance-matched, the energy of the two frequency bands is rectified separately, and finally, the DC energy is output to the DC module. However, since the output impedances ($Z_{\mathrm{out}}$) of the two frequency bands are not inherently correlated, the impedance transformations need to be realized through a specific microstrip line structure to make them satisfy the conjugate matching relationship:

(2) $$\left\{\begin{array}{l} Z_{\mathrm{out}}=R_{\mathrm{out}}+jX_{\mathrm{out}}@f1\\ Z_{\mathrm{out}}^{*}=R_{\mathrm{out}}-jX_{\mathrm{out}}@f2 \end{array}\right.$$

The implementation is divided into two steps. Resistance matching: priority is given to the microstrip line structure to adjust the real part ($R_{\mathrm{out}}$). Reactance matching: the compensation of the imaginary part ($X_{\mathrm{out}}$) is performed by means of a π-network. This hybrid matching scheme significantly reduces the design complexity compared to purely aggregate element networks. The design of the microstrip line structure is based on the transmission line theory, where the relationship between the input impedance ($Z_{in}$) and the load impedance ($Z_{L}$) is described by the transmission line equation:

(3) $$Z_{in}=Z_{0}\frac{Z_{L}+jZ_{0}tan\beta l}{Z_{0}+jZ_{L}tan\beta l}$$

(4) $${\left.Z_{\mathrm{out}}\right|}_{f_{1,2}}=\left(Z_{0}\right)\frac{{\left.Z_{\mathrm{in}}\right|}_{f_{1,2}}-jZ_{0}tan\theta_{0}}{Z_{0}-{\left.jZ_{\mathrm{in}}\right|}_{f_{1,2}}tan\theta_{0}}$$

In the transmission line equation, Equation (4), $Z_{0}$ is the microstrip line characteristic impedance; βl is the electrical length; $Z_{L}$ represents the load impedance; and $Z_{in}$ represents the input impedance. The output impedance (${\left.Z_{\mathrm{in}}\right|}_{f_{1,2}}$) of the two frequency bands ($f_{1,2}$) is also calculated separately by Equation (5). The impedance of the two frequency bands needs to meet the conjugate matching requirements $\left({\left.Z_{\mathrm{out}}\right|}_{f_{1,2}}=R_{1,2}+X_{1,2}\right)$, furthermore, the impedance equation can be extrapolated to Equation (6), where n is an integer.

(5) $$Z_{0}=\sqrt{R_{1}R_{2}+X_{1}X_{2}+\frac{X_{1}+X_{2}}{R_{2}-R_{1}}\left(R_{1}X_{2}-R_{2}X_{1}\right)}$$

(6) $$\begin{array}{rr} & \beta l=\frac{n\pi +arctan\left(\frac{Z_{0}\left(R_{1}-R_{2}\right)}{R_{1}X_{2}-R_{2}X_{1}}\right)}{1+r} \end{array}$$

The impedance value of the microstrip line is thus calculated, and its line width (w) at (*w*/*h* < 1) can be calculated by the following, Equation (7):

(7) $$\begin{array}{ll} & R_{\mathrm{out}}=\frac{60}{\sqrt{\varepsilon_{\mathrm{eff}}}}ln\left(\frac{8h}{w}+\frac{w}{4h}\right)\\ & \varepsilon_{\mathrm{eff}}=\frac{\varepsilon_{r}+1}{2}+\frac{\varepsilon_{r}-1}{2}\left\{{\left[1+12\frac{h}{w}\right]}^{-0.5}+0.04\left[1-\frac{w}{h}\right]\right\} \end{array}$$

where $\varepsilon_{r}$ is the substrate relative dielectric constant, h is the substrate height, and $Z_{0}\;$ is the characteristic impedance. After taking actual measurements of the antenna designed in Section 3, the actual impedance of the prepared antenna is given in Equation (8) below:

(8) $$Z_{\mathrm{in}}(\Omega )=\left\{\begin{array}{ll} 85+j50 & @f_{1}\\ 102-j72 & @f_{2} \end{array}\right.$$

Based on the above data and methodology, a π-matching network was designed for two frequency bands at $f_{1}$ = 915 MHz and $f_{2}$ = 2.45 GHz, respectively, and the known thickness h of the dielectric substrate, the relative permittivity er, and the characteristic impedance $Z_{0}$ can be calculated from equations. The line width of the microstrip line is calculated from Equation (9), and furthermore, based on the obtained width of the microstrip line, w, the length of the microstrip line, L, is obtained by using Equation (10), with the actual data of the microstrip line obtained from further simulation and optimization by using ADS simulation software shown below in Table 2.

(9) $$\begin{array}{ll} & \frac{w}{h}=\frac{8e^{A}}{e^{2A}-2}\\ & A=\frac{Z_{0}}{60}\sqrt{\frac{\varepsilon_{r}+1}{2}}+\frac{\varepsilon_{r}-1}{\varepsilon_{r}+1}\left(0.23+\frac{0.11}{\varepsilon_{r}}\right) \end{array}$$

(10) $$L=\frac{c}{f\sqrt{\varepsilon_{eff}}}\times \frac{\theta_{0}}{2\pi }$$

**Figure 7:**
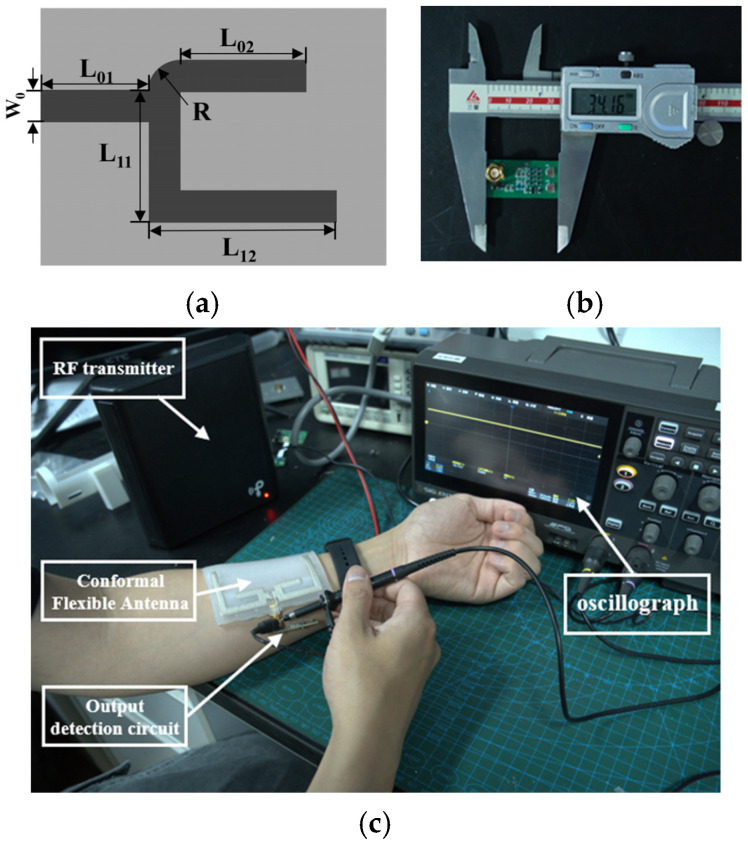
(**a**) Schematic diagram of microstrip structure; (**b**) output monitoring circuit; (**c**) experimental test scenarios.

The branching structure of the matching circuit microstrip is shown in Figure 7a, where L_01_ + L_02_ + R = $l_{0}$ (f = 915 MHz) and L_01_ + L_11_ + L_12_ = $l_{0}$ (f = 2.45 GHz), after which the π-type matching circuit needs to match only the imaginary parts of Z_1_ and Z_2_. L_01_ = 152.27 mil; L_02_ = 195.29 mil; L_11_ = 289.37 mil; and L_12_ = 348.33 mil.

The full-bridge rectifier circuit topology exhibits lower requirements for lumped capacitive elements. Owing to its symmetrical structure, it efficiently harvests both positive and negative half-cycles of RF energy, demonstrating superior power conversion efficiency and enabling higher power extraction [25]. Therefore, this work adopts the full-bridge rectifier architecture to obtain more stable and sustainable DC energy.

Since the peak voltage of the AC signal received from the antenna is typically significantly lower than the diode’s threshold voltage, diodes with low turn-on thresholds are essential. Furthermore, as the energy harvesting circuit operates at high frequencies, the diodes must also feature fast switching characteristics [26]. To address these requirements, the B5819WS Schottky diode is selected in this design, which exhibits an exceptionally low turn-on voltage of merely 150 mV, satisfying the operational conditions.

A full-wave bridge rectifier is implemented using low forward-voltage Schottky diodes (B5819WS, CJ) and smoothing capacitors. The rectified energy is stored in a supercapacitor (KORCHIP, SM3R3333T01) and subsequently regulated to 3.3 V through a 3.3 V Zener diode (JSMSEMI, MM3Z3V3BW) and an ultra-low-power Low-Dropout Regulator (LDO) (SGMICRO, SGM2040-3.3YN5G/TR). The regulated output is delivered to a load matched with the system impedance. The designed dual-band energy output detection circuit is illustrated in the accompanying Figure 7b. Its electrical wiring diagram can be seen in Supplementary Materials Figure S1.

This paper presents a theoretical and experimental investigation of the power transfer characteristics in the above dual-band wireless energy transmission system. The power transfer model is established based on the Friis free-space path loss formula, where the received power $P_{r}$ is determined by the transmitted power $P_{t}$, antenna gains $G_{t}$ and $G_{r}$, operational wavelength λ, and transmission distance r according to Equation (11), with the far-field boundary calculated in Equation (12) and determined to be 4 cm.

(11) $$P_{r}=P_{t}G_{t}G_{r}{\left(\frac{\lambda }{4\pi r}\right)}^{2}$$

(12) $$r_{far-field}=\frac{2D^{2}}{\lambda }$$

**Figure 8:**
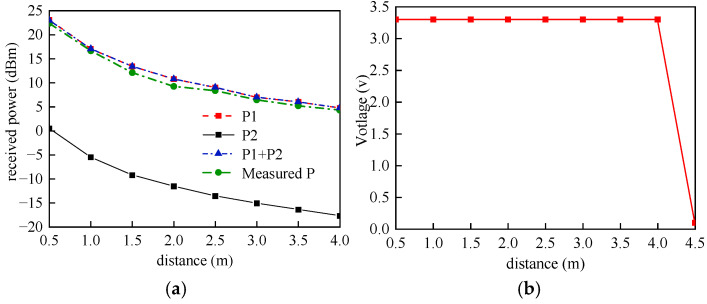
(**a**) Received power versus distance; (**b**) experimental test scenarios.

The experimental setup employs a dual-band configuration operating at 915 MHz (3 W, 8 dBi) and 2.45 GHz (100 mW, 9 dBi), coupled with a flexible receiving antenna providing 3.3 dBi gain. Through comparative analysis between system simulations and experimental measurements, the power transfer characteristics at various transmission distances were obtained, as shown in Figure 8 below.

As illustrated in Figure 8a, P_1_ represents the simulated received power under 915 MHz single-band operation, while P_2_ denotes the simulated received power under 2.45 GHz single-band operation. The combined simulated received power (P_1_ + P_2_) accounts for simultaneous dual-band (915 MHz/2.45 GHz) energy transmission. Experimental measurements (denoted as Measured P) under dual-band conditions are included for comparative analysis. The relationship between output power, output voltage, and transmission distance reveals that the energy harvesting system maintains optimal performance within a 4 m range. Notably, the 2.45 GHz contribution (P_2_) exhibits significantly lower-power magnitude compared to the 915 MHz component (P_1_), resulting in minimal impact on the overall system reception performance. This characteristic suggests that the 915 MHz band dominates the power reception characteristics in the dual-band operation mode.

## 5. Human Physical Parameter Monitoring System

**Figure 9:**
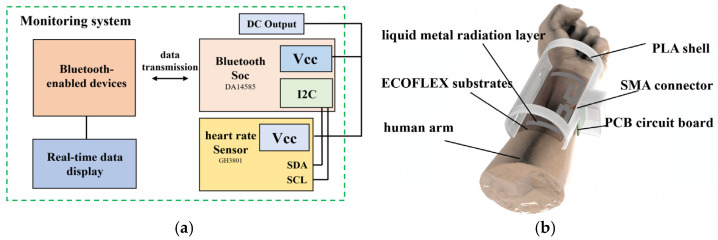
(**a**) Monitoring system components; (**b**) wearable system components.

Figure 9 illustrates the architecture of the complete wearable system, which consists of the following components: a flexible receiving antenna designed to harvest dual-band (915 MHz/2.45 GHz) electromagnetic energy; a dual-band rectifier circuit that converts the RF energy into stable DC output; and a physiological monitoring system (Figure 9a) powered by the harvested energy. In this study, we developed a low-power monitoring system with the following operational workflow: The DC power generated by the energy harvesting module drives a main control unit, which acquires multi-channel physiological sensor data through high-precision ADC sampling. The data is then wirelessly transmitted via a Bluetooth Low Energy SoC to a mobile terminal. A dedicated application on the terminal device provides real-time visualization of the monitored parameters. Figure 9b presents an exploded-view schematic of the physical system, detailing the layered structure including the liquid metal radiation layer, PCB circuit, SMA connector, and PLA encapsulation design. The system employs a flexible substrate, with all functional modules interconnected through microconnectors to achieve high-density integration. This design ensures both wearer comfort and signal integrity while maintaining robust performance.

The monitoring system employs a low-power microcontroller (DA14585, Renesas, Beijing, China) with integrated Bluetooth Low Energy (BLE) functionality for wireless data communication. For physiological signal acquisition, an optical heart rate sensor (GH3018, Goodix, Shenzhen, China) is implemented, which interfaces with the main controller through an Inter-Integrated Circuit (I^2^C) serial communication bus (including Serial Data Line (SDA) and Serial Clock Line (SCL)). Data transmission is achieved via a chip antenna (ANT1608LL14R2400A, Yageo, New Taipei City, Taiwan) operating under the BLE protocol, enabling real-time heart rate monitoring on paired Bluetooth devices.

**Figure 10:**
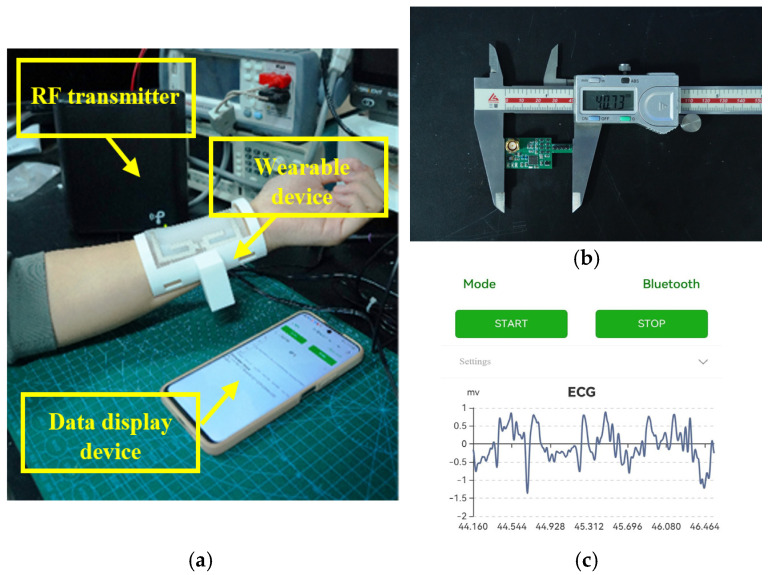
(**a**) Physiological monitoring system schematic; (**b**) physical implementation of the wearable physiological monitoring system circuit board; (**c**) physiological monitoring system user interface.

Figure 10b presents the physical implementation of the monitoring system’s printed circuit board (PCB), Its electrical wiring diagram can be seen in Supplementary Materials Figure S2. Power consumption measurements using a constant power analyzer indicate that the system operates at approximately 60 mW (18 dBm) during steady-state operation. Based on the power transfer analysis presented in the preceding section, the operational range of the monitoring system is constrained within 1 m to ensure reliable performance. Based on the experimental protocol illustrated in Figure 10a, we conducted a comprehensive performance evaluation of the wearable monitoring system.

System validation within this operational range demonstrates successful real-time data acquisition and transmission. The graphical user interface of the paired device displays continuous pulse waveform data, as shown in the accompanying figures. Figure 10c specifically illustrates the wearable device operating within its effective energy harvesting range, with the Bluetooth interface providing persistent and reliable heart rate monitoring.

These experimental findings validate the system’s conformance to design specifications and demonstrate its clinical applicability, particularly noting exceptional performance in energy utilization efficiency and signal quality—critical factors for subsequent product development. An introductory demonstration of the entire device is shown in Video S2.

## 6. Conclusions

This study proposes a conformal 3D printing process for fabricating flexible antennas with curved surfaces that conform to the human wrist, significantly reducing performance degradation during bending deformation. Through multi-material heterogeneous printing that alternately deposits conductive silver paste and flexible ECOFLEX encapsulation layers, dual-band impedance matching at 915 MHz/2.45 GHz was achieved. This provides a novel solution for manufacturing conformal wearable receiving antennas. A corresponding dual-band energy harvesting circuit and environmental monitoring system were designed to validate the feasibility of wireless power transfer, advancing the development of wireless power supply devices. However, the current system still has the following limitations: The environmental monitoring module utilizes rigid FR-4 PCB substrates, compromising wearability due to mechanical constraints; the stable operating charging range remains limited by energy harvesting capacity and system complexity.

Future work will focus on developing flexible hybrid electronic systems and optimizing antenna structural design to further extend the effective charging range.

## Figures and Tables

**Table 1:** Conformal antenna design parameters.

Parameters	Value (mm)	Parameters	Value (mm)
L	90	L2	21
W	50	W2	5
L1	35	H	3
W1	34	h	1
lineW	5	d	3

**Table 2:** Conformal antenna design parameters.

f (Ghz)	$w_{0}$ (mil)	$l_{0}$ (mil)
0.915	37.12	483.17
2.45	37.12	926.48

## Data Availability

The original contributions presented in this study are included in the article. Further inquiries can be directed to the corresponding author.

## Supplementary Materials

The following supporting information can be downloaded at: https://www.mdpi.com/article/10.3390/s25144488/s1, Figures S1 and S2: Electrical wiring diagrams; Video S1: Print preparation process; Video S2: Introductory demonstration of the entire device.
