# Supplementary figures and images for: Flexible Receiver Antenna Prepared Based on Conformal Printing and Its Wearable System
Source: Sensors (Basel). 2025 Jul 18;25(14):4488. doi: 10.3390/s25144488 (PMC12300397; doi:10.3390/s25144488)

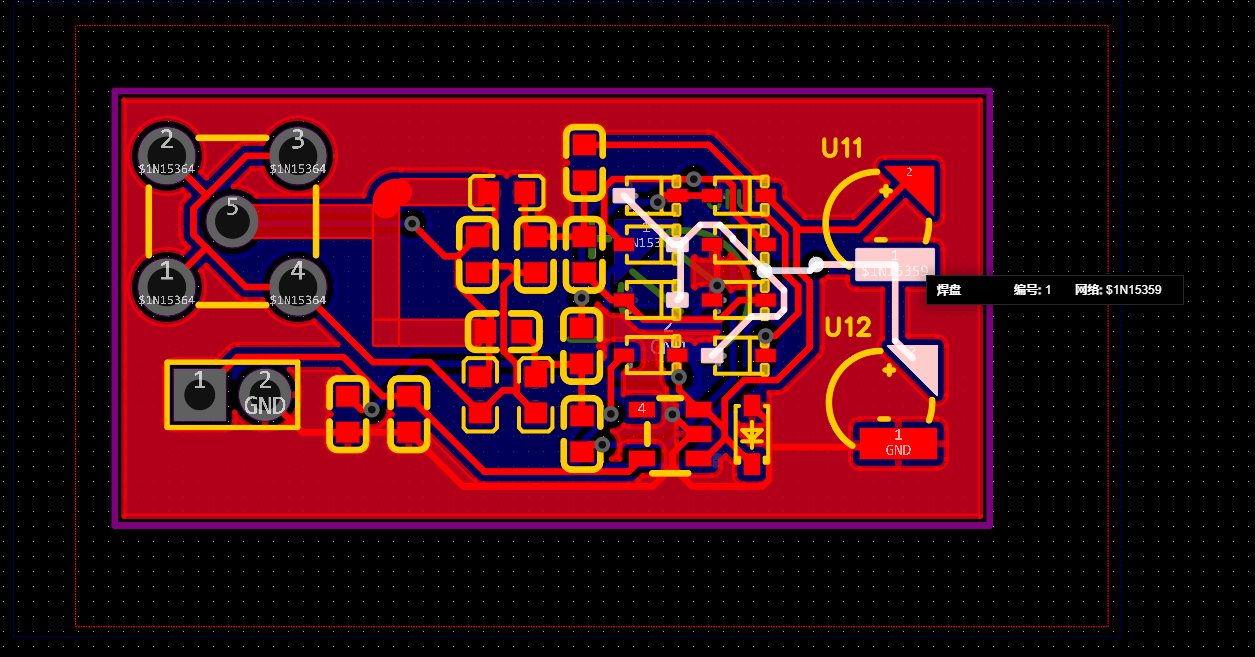

Supplement: Supplementary file 1 [file sensors-25-04488-s001.zip › Figure S1 Electrical wiring diagram.png]

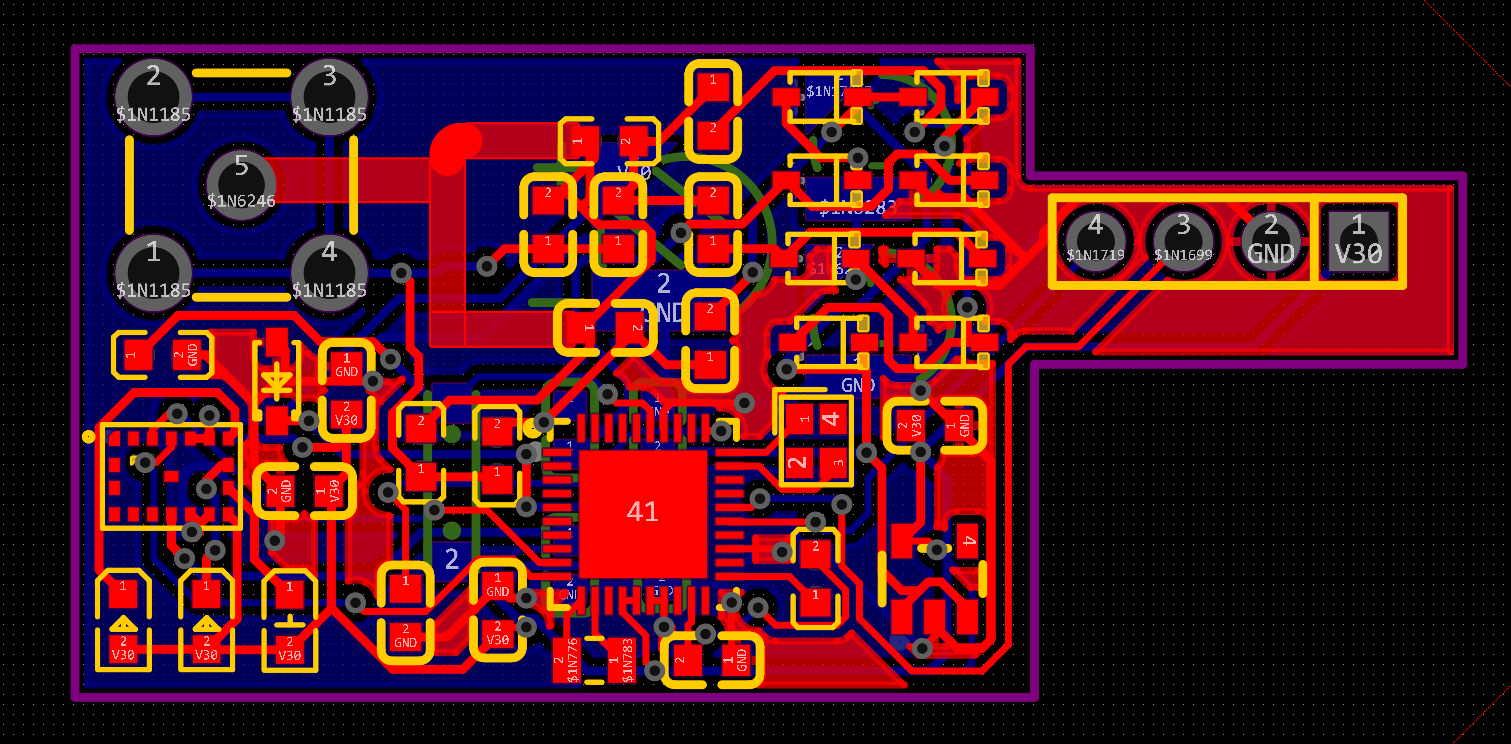

Supplement: Supplementary file 1 [file sensors-25-04488-s001.zip › Figure S2 Electrical wiring diagram.png]
